# Brain structural correlates of upward social mobility in ethnic minority individuals

**DOI:** 10.1007/s00127-021-02163-0

**Published:** 2021-08-12

**Authors:** Janina I. Schweiger, Necip Capraz, Ceren Akdeniz, Urs Braun, Tracie Ebalu, Carolin Moessnang, Oksana Berhe, Zhenxiang Zang, Emanuel Schwarz, Edda Bilek, Andreas Meyer-Lindenberg, Heike Tost

**Affiliations:** 1 Department of Psychiatry and Psychotherapy, Central Institute of Mental Health, Medical Faculty Mannheim, University of Heidelberg, Square J5, 68159 Mannheim, Germany; 2 Department of Psychology, Istanbul Gelisim University, Istanbul, Turkey

**Keywords:** Socioeconomic status, Anterior cingulate gyrus, Gray matter, Stress, Mental health

## Abstract

**Purpose:**

Perigenual anterior cingulate cortex (pACC) is a neural convergence site for social stress-related risk factors for mental health, including ethnic minority status. Current social status, a strong predictor of mental and somatic health, has been related to gray matter volume in this region, but the effects of social mobility over the lifespan are unknown and may differ in minorities. Recent studies suggest a diminished health return of upward social mobility for ethnic minority individuals, potentially due to sustained stress-associated experiences and subsequent activation of the neural stress response system.

**Methods:**

To address this issue, we studied an ethnic minority sample with strong upward social mobility. In a cross-sectional design, we examined 64 young adult native German and 76 ethnic minority individuals with comparable sociodemographic attributes using whole-brain structural magnetic resonance imaging.

**Results:**

Results showed a significant group-dependent interaction between perceived upward social mobility and pACC gray matter volume, with a significant negative association in the ethnic minority individuals. Post-hoc analysis showed a significant mediation of the relationship between perceived upward social mobility and pACC volume by perceived chronic stress, a variable that was significantly correlated with perceived discrimination in our ethnic minority group.

**Conclusion:**

Our findings extend prior work by pointing to a biological signature of the “allostatic costs” of socioeconomic attainment in socially disadvantaged upwardly mobile individuals in a key neural node implicated in the regulation of stress and negative affect.

**Supplementary Information:**

The online version contains supplementary material available at 10.1007/s00127-021-02163-0.

## Introduction

Social-economic inequality strongly affects physical and mental health outcomes at every level of the social hierarchy [1–3]. Specifically, perceived social status, or a person's understanding of his or her relative social status in society, affects physical and mental health outcomes above and beyond objective criteria of socioeconomic status [4, 5]. In this context, “perceived social status mobility” refers to the sense of a person’s change in social status within a given social hierarchy from early life and can be quantified by the extent to which the perceived social status of an adult deviates from that of the birth family during early childhood.

Health effects of social status mobility are difficult to generalize and may vary depending on the affiliation to a respective population group [6]. Besides noticeable economic and educational changes, social status mobility—independent of its direction—can be accompanied by shifts in social networks and social support structures that require behavioral flexibility. While the loss of social status can have deteriorating physical and mental health implications [7] recent studies raised awareness of the influence of upward social mobility and its possible attendant effects on health [8].

Upward social mobility enables individuals to reach favorable educational attainments, increases in income and has been linked to a lower prevalence of health risks [9] and increased well-being [10]. However, the benefits of upward social mobility do not always translate into gains in physical health and psychological well-being [11, 12]. Several lines of evidence suggest that this may be especially so for people from ethnic minorities. For example, in children of first-generation immigrants, a minority group at increased risk for psychiatric illness [13], climbing the social ladder in a majority society of different ethnic origin comes with a multitude of potential social stressors including lack of parental capital, lack of specific role models, discrimination experiences, pressure to acculturate to the “elite habitus” [14] and distancing from established social support structures [15, 16]. These adverse social factors may plausibly converge on increased social stress exposure [17, 18], an established risk factor for physical and mental health, and may outweigh the benefits of upward social mobility on well-being seen in their ethnic majority peers [7, 19, 20].

The processing of subjective social status at the neural system level is mediated by limbic brain regions, including anterior cingulate cortex (ACC), amygdala and hippocampus [21, 22]. These regions are commonly associated with the detection and evaluation of social experiences and cues, regulation of emotion responses, and control of physiological stress responses [23, 24]. Adverse experiences in early life profoundly influence brain developmental plasticity in these regions [25], particularly in ACC [26, 27]. Consistent with this, decreased gray matter volume in perigenual ACC (pACC) has been repeatedly associated with measures of social status [28] including lower levels of education [29], lower perceived social status [30] and lower childhood socioeconomic status [31]. These and other findings [32–36] motivated the hypothesis that pACC is likely a neural convergence site for early social stress-related risk factors for mental health in the brain [37, 38].

Despite these links between social stress in early life, mental health risk and pACC volume, the brain structural correlates of perceived upward social mobility are unexplored to date. The inclusion of social upwardly mobile descendants of first-generation immigrants in such a study offers the opportunity to examine whether ethnic minority individuals show a different association between perceived upward social mobility and pACC volume compared to their ethnic majority peers, as suggested by the presumed increase in social stress exposure while climbing the social ladder in a majority society of different ethnic origin during vulnerable periods of brain development. In a cross-sectional design, the current study thus aimed to explore (1) the potential effects of upward social mobility on pACC morphology in ethnic minority and native German individuals, thereby investigating the neural correlates of social status changes in different subpopulations. Given that increased pACC gray matter volume has been related to higher social standing [30] in a majority ethnic group (i.e., Americans of European descent), we expect to see an inverse pattern in the descendants of first-generation immigrants, i.e., lower pACC volume among upwardly mobile individuals with an ethnic minority status. (2) We further hypothesized the presence of higher chronic stress levels among ethnic minority individuals that have successfully climbed the social ladder in a majority society of different ethnic origin, and (3) that perceived chronic stress mediates the relationship between upward social mobility and pACC volume.

## Methods and materials

### Use of terms

We decided to use the term “ethnic minority individuals” in this publication. We decided against the term “second-generation immigrant”, an expression widely used in Germany to describe Germans born to Migrant-Parents because this work does not focus on the effects of migration or peri-migration processes (e.g., post-migration stress, refugee trauma etc.) but on the perceived social adversity experienced as an individual separated from the majority society as mechanisms of action.

### Participants

A total of 140 right-handed, healthy young volunteers (43 men and 97 women; mean age = 23.06; SD = 4.04, age range: 18–46) participated in this study. Participants were residing in communities in and surrounding the city of Mannheim in the south-western part of Germany. For recruitment, we used several strategies such as newspaper advertisement, information from local registration offices, flyers, posters, and internet platforms. Sixty-four participants were native Germans (i.e., both parents were of German origin), and 76 participants had a non-German ethnic background (i.e., both parents were born in a foreign country and had moved to Germany). The majority (63 participants, 82.9%) of our ethnic minority sample were “second-generation immigrants” that were born in Germany to non-German parents. Thirteen Individuals (17.1%) of our ethnic minority sample had moved to Germany from a foreign country up to 5 years of age. Individuals with an ethnic minority status consisted migrants from various backgrounds: 54 (71%) individuals with Turkish-European lineage, 17 (22.3%) individuals with other European ancestries (e.g. Poland, Rumania, Russia), 4 (5.2%) individuals with Middle Eastern/African, and 1 (1.3%) individual with an East-Asian background. Characteristics of the samples are provided in Table 1. All participants provided written informed consent to a study protocol approved by the institutional review board of the University of Heidelberg. Exclusion criteria included a lifetime history of general medical, psychiatric or neurological illness, prior psychopharmacological or psychotherapeutic treatment, drug or alcohol abuse, MR contraindications, and a history of head trauma. As most neuroimaging work is preselected due to MRI limits, this implicates that we cannot claim full generalizability our findings to the general population.

**Table 1:** Sample description of participant groups

	Germans		Ethnic minority group		Group comparison
	*M* ± SD/count	*n*	*M* ± SD/count	*n*	*p* value
Demographic data					
Sex (male/female)	19/45	64	24/52	76	0.855
Age (years)	23.48 ± 3.80	64	22.71 ± 4.22	76	0.256
Education (years)	12.34 ± 1.29	64	12.40 ± 1.05	76	0.747
Smokers (non-smokers/smokers)	51/13	64	51/25	76	0.127
BMI: mean (kg/m^2^)	22.94 ± 3.37	64	22.97 ± 3.42	76	0.951
Marital status (single/married)	58/5	63	68/8	76	0.772
Current employed (not working/working)	24/40	64	25/51	76	0.597
Household size (number of persons)	2.71 ± 2.15	64	3.47 ± 1.81	76	0.028*
Household income (€)	1805 ± 1494	64	2069 ± 1593	76	0.314
Current urbanicity	2.66 ± 0.59	64	2.79 ± 0.52	76	0.167
Early-life urbanicity	35.13 ± 9.23	64	36.57 ± 9.75	76	0.372
Psychological data					
Perceived social status (McArthur scale, self)	6.38 ± 1.58	64	6.50 ± 1.33	76	0.619
Perceived social status (McArthur scale, parents)	6.28 ± 1.84	64	4.13 ± 1.61	76	0.000**
Perceived social mobility (McArthur scale, self-parents)	0.09 ± 1.90	64	2.36 ± 1.90	76	0.000**
Chronic stress (CSSS)	13.78 ± 5.88	42	20.18 ± 8.94	65	0.000**
Social Support (BSSS)	3,86 ± 0.79	54	3.68 ± 0.45	76	0.153
Perceived Self- Discrimination	–	–	2.32 ± 0.96	66	–
Perceived Group-Discrimination	–	–	3.48 ± 1.01	66	–

*Significant at *p* < 0.05

**Significant at *p* < 0.001

### Psychological assessments

*Perceived current social status* was measured using an adapted version of the McArthur Scale of Subjective Social Status [1, 30] (http://www.macses.ucsf.edu/) which we translated from the original English version to German. The “social ladder” is a visual analog scale (VAS) depicting a ladder with ten rungs. Rung 1 of the ladder represents the lowest level of perceived own social standing, while rung 10 represents the highest possible level of self-ascribed social status. To determine the perceived current social standing of the participants, we asked participants to indicate the rung that best represented their own current “standing” relative to that of other individuals living in the German society based on income, education, and occupational status. In addition, to obtain a measure of the *perceived social status of the birth family*, we also asked them to rank the perceived social status of their parents at the time of the participant’s birth. We used a second, but otherwise identical, “social ladder” VAS for this purpose. Here, we asked all participants to indicate the rung that best represented where their parents “stood,” relative to that of other individuals living in German society, when the participant was born. Details of the perceived own current social status (own social status) and the status of the family of origin at the time of the participant’s birth (birth family status) of the study groups are given in Table 1. In addition, for each individual, we calculated a *“social status mobility” score* capturing the difference between the current perceived social status and the birth family status scores (see Table 1 for details).

*Perceived chronic stress* was measured with the *Chronic Stress Screening Scale* (CSSS), which is based on Trier Inventory for the Assessment of Chronic Stress (TICS) [39]. The CSSS consists of 12 items (e.g., “Even though I gave my best, my work is not good enough”) assessing the chronic stress level in the past three months through 5-point Likert scales (from “0” = never, to “4” = very often). Internal consistency (Cronbach α) of the scale was reported as 0.91 [39]. Measures of *perceived social support* were assessed using a subscale of the Berlin Social Support Scale (BSSS). The scale consists of 8 items measuring cognitive and behavioral aspects of social support, as well as the type of social support available under normal and stressful life circumstances with four-point Likert scales. The internal consistency of this subscale was reported as α = 0.83 [40]. For participants with an ethnic minority status, we further assessed *perceived discrimination* of the individual and of his (or her) own ethnic group in German society using an adapted version of the perceived discrimination scale of Ruggiero and Taylor [41] as described previously in detail [32].

### MRI data acquisition

Structural MRI was performed on a 3-Tesla Siemens Magnetom Tim Trio (Siemens, Erlangen, Germany) system located at the Central Institute of Mental Health, Mannheim. We used a T1-weighted 3D magnetization-prepared rapid gradient-echo (MPRage) sequence with whole-brain coverage, an isotropic spatial resolution of 1 mm^3^ and the following sequence specifications: TR = 2300 ms (ms), TE = 3.03 ms, flip angle = 9°, 192 contiguous sagittal slices, 1-mm slice thickness, field of view = 256 mm.

### Image processing

Images were processed using the VBM toolbox (VBM8, http://dbm.neuro.uni-jena.de/vbm) implemented in the Statistical Parametric Mapping (SPM) software, (SPM8; Welcome Trust Centre for Neuroimaging, University College London, UK; http://www.fil.ion.ucl.ac.uk/spm) running in MATLAB R2011a (MathWorks, Natick, MA). We used default parameters as described in the VBM8 toolbox manual. Voxel-based morphometry (VBM) is an automated method allowing for the processing of whole-brain structural MR images and the quantification of voxel-wise gray matter volumes [42]. Briefly, image processing included tissue classification into gray matter, white matter, cerebrospinal fluid, and three other non-cerebral tissue classes, normalization to Montreal Neurological Institute (MNI) space with a diffeomorphic image registration algorithm (DARTEL), correction for image intensity non-uniformity, a thorough cleaning up of GM partitions, the application of a hidden Markov random field model, and spatial adaptive nonlocal means de-noising. The resulting tissue segments were multiplied by the Jacobian determinants of the deformation field to transform the GM density values into volume equivalents and corrected for individual total intracranial volume. The segmented, normalized, noise-corrected, and modulated GM images were then smoothed with a 10-mm full width at half maximum isotropic Gaussian kernel.

### Analysis of psychological measures

Data analysis of the psychological and demographic measures was performed using the SPSS Predictive Analytics Software (SPSS 24, IBM Inc., Armonk, New York, USA). We used Chi-square tests to examine group differences for categorical variables and *t*-tests for independent samples to examine group differences for continuous variables. The level of statistical significance for psychological variables was defined as *p* < 0.05 (two-tailed).

### Associations between perceived social mobility and brain gray matter volume

Across all participants, we defined a multiple regression model with perceived social mobility as covariate of interest, gray matter volume maps as the dependent variable, and variables for basic demographics (i.e., age, gender), objective social status (i.e., education, monthly income, employment status), urbanicity (i.e., early life urbanicity, current urbanicity) and household size as confounder. The possible interaction of perceived social mobility with ethnic minority background was tested using an ANOVA model including group (i.e., Germans, ethnic minority group) as a factor and a “group by perceived social mobility” interaction term as effect of interest. In the interaction analysis we used the same covariates as described for the perceived social mobility regression model.

### Multiple comparisons correction in imaging space

Consistent with prior work from our own laboratory [32–35] and other groups [30], we expected social risk-associated structural effects mapping to the pACC, a crucial neural node for the top-down regulation of neural responses in emotion and stress processing circuitries [22]. To reflect this a priori hypothesis, all imaging analyses were family-wise error (FWE) corrected for multiple comparisons for a region of interest (ROI) covering the rostral ACC. As in our prior work, we used an anatomical mask from the Harvard Oxford Atlas (HO, http://www.cma.mgh.harvard.edu) which we modified to cover the rostral-ventral affective divisions of the ACC as defined by Bush and colleagues [43] or the areas pACC and sgACC in the nomenclature used by Etkin and colleagues [44] (see [32] for details).

### Post-hoc mediation analysis

**Fig. 1:**
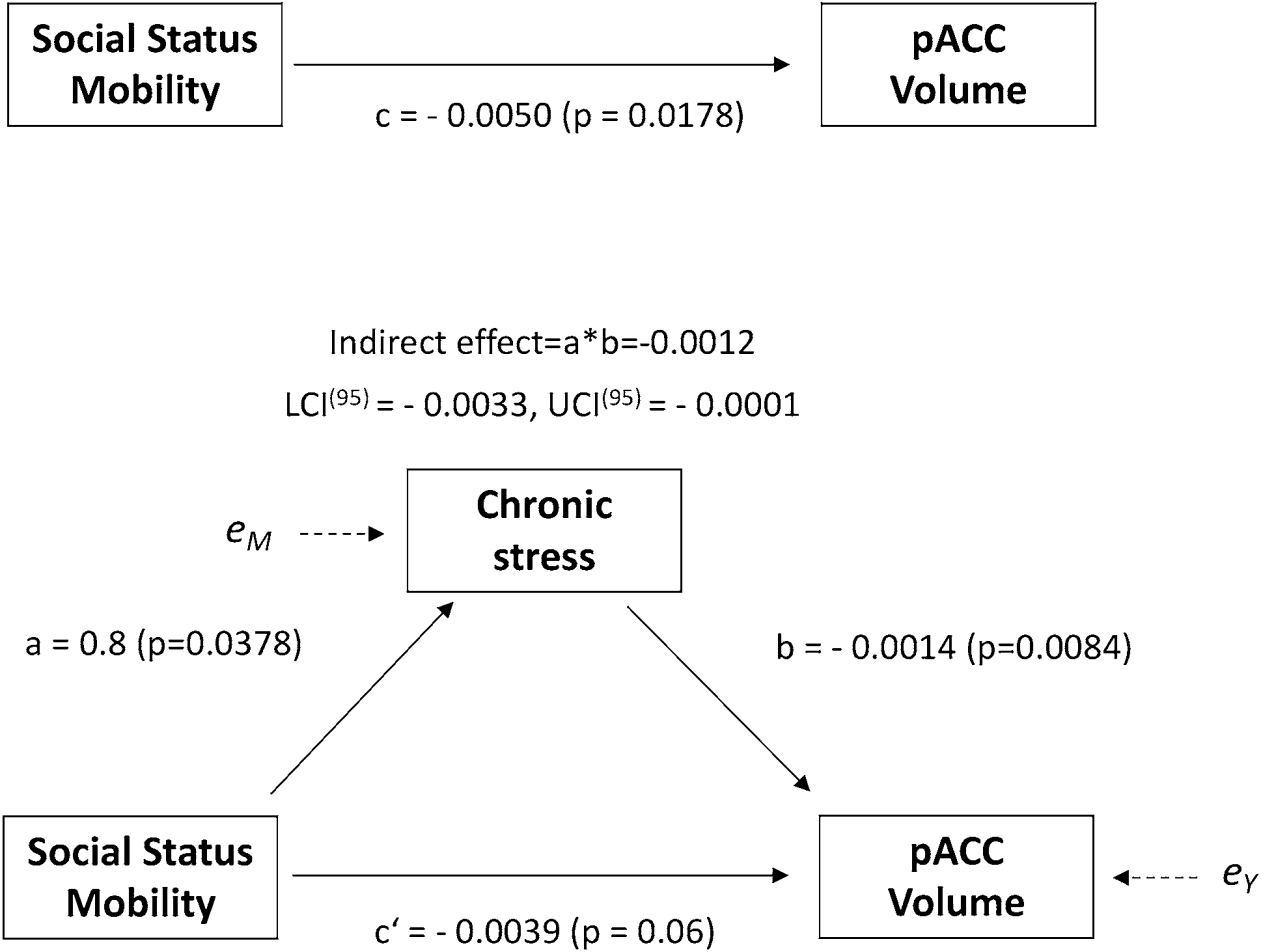
The statistical model of mediation analysis. The effect of a casual variable (*X*) on an outcome variable (*Y*) through a mediator variable (*M*) can be tested by up to ten covariates. Variable *M* is a mediator if *X* significantly predicts *M* (Path *a*), X significantly predicts in *Y* path (Path *c*; representing the total effect), *M* significantly predicts in *Y* (Path *b*) when controlling for *X*, and the effect of *X* on *Y* reduces significantly when *M* as well as *X* simultaneously predicts *Y* (Path *c*; representing the direct effect)

A plausible intermediate between perceived social status mobility and altered brain structure in adulthood is chronic social stress. We explored this hypothesis with a mediation analysis using the SPSS macro PROCESS [45]. PROCESS estimates the indirect effects of a proposed causal variable on an outcome variable through a mediator, thereby controlling for confounders. We defined perceived social status mobility as the proposed independent variable (*X*), mean pACC gray matter volume (extracted from the pACC mask, see above) as the outcome variable (*Y*), and chronic stress estimates (CSSS) as the proposed mediator (*M*). This model therefore tests whether the effect of perceived social mobility on pACC volume is mediated by chronic stress. Since chronic stress scores were only available for 107 participants (*n* = 42 Germans, *n* = 65 ethnic minority individuals, see Table 1) we conducted the post-hoc mediation analysis for these participants. We further included age, gender, education, employment status, monthly income, early life urbanicity, current urbanicity and household size as confounder. In a second step, we explored the possibility of a group-specific mediation effect by extending the same mediation model by a proposed dichotomous moderator variable (i.e., German vs. ethnic minority group) influencing the relationship between perceived social status mobility and chronic stress (or the “a” path in the mediation model). For non-parametric statistical inference, we calculated bias-corrected 95% percentile bootstrap confidence intervals (CIs) based on 10,000 bootstrap samples. An illustration of the statistical models is given in Fig. 1*.*

## Results

### Psychological measures

Details on the acquired demographic and psychological measures are provided in Table 1. Both groups of individuals were comparable for all demographic variables (*p* values > 0.100) except for household size (*p* = 0.028), which was larger among the ethnic minority individuals. Current perceived social status did not differ between groups (*p* = 0.619). However, the perceived social standing of the family of origin at the time of the participants' birth was significantly lower in the ethnic minority group (*p* < 0.001, see Table 1). Moreover, the ethnic minority group had significantly higher social mobility scores (*p* < 0.001) and reported higher chronic stress levels (CSSS, *p* < 0.001) than the German participants. In individuals with ethnic minority background, higher chronic stress levels correlated positively with perceived self (*β* = 0.29, *p* = 0.017) and ethnic group discrimination (*β* = 0.39, *p* = 0.001) (see Supplementary material).

### Brain volumetric correlates of upward social mobility

**Fig. 2:**
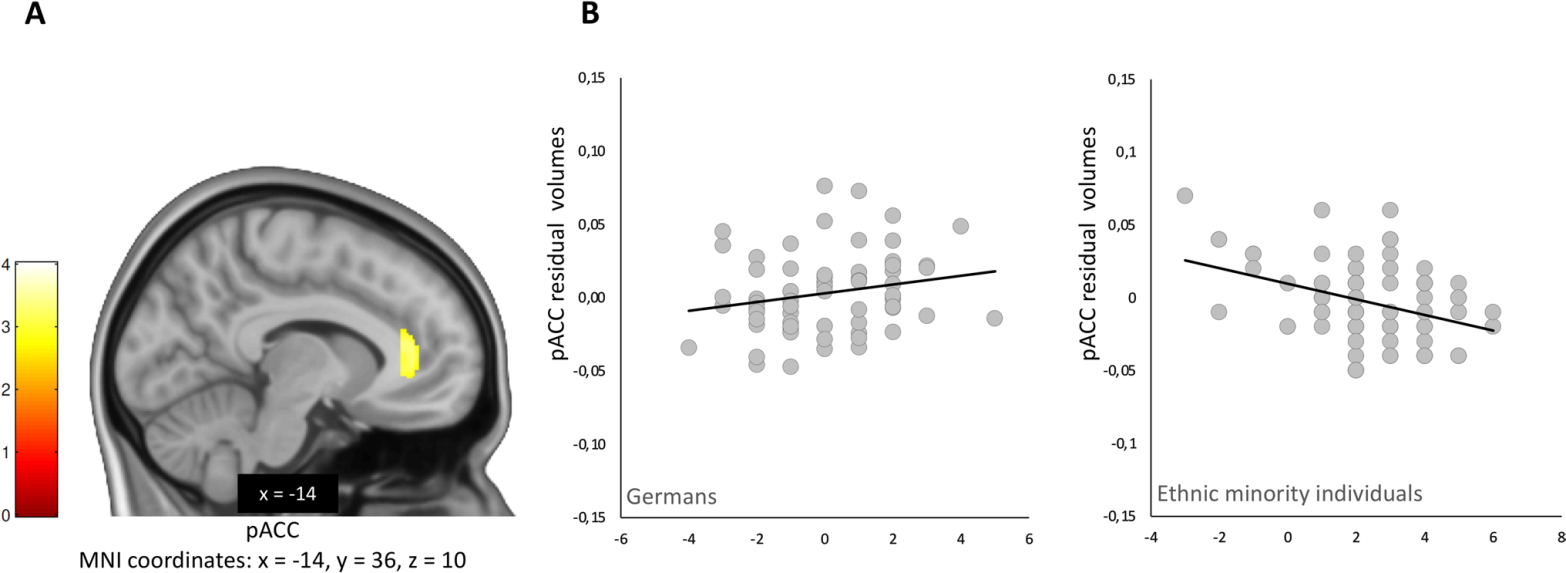
**A** Effect of social status mobility scores by group interaction displayed at *p* < 0.005; **B **Scatterplot of pACC volumes (residual volumes extracted from peak voxel cluster) within the mask interacting with social status mobility scores for Germans and Ethinic scores for minority individuals, respectively

There was no significant main effect of social mobility on pACC volume across study groups. However, the group by social mobility interaction analysis provided evidence for a significant group-dependent difference in the association between the perceived social mobility of individuals and pACC volume (MNI: *x* = −14, *y* = 36, *z* = 10; *T* = 3.54, *p*_FWE_ = 0.026, ROI corrected, see Fig. 2A). Post-hoc analysis in both groups separately showed no significant association between upward social mobility in the German majority group, but a significant negative association in the ethnic minority group (MNI *x* = −12, *y* = 35, *z* = 13; *T* = 3.61, *p*_FWE_ = 0.030, ROI corrected). The slope of the association suggested that ethnic minority participants with a higher perceived upward social mobility had lower gray matter volumes in pACC (Fig. 2B).

Post-hoc analysis within the group of ethnic minority individuals showed no significant interaction between the group of ethnic minority individuals born in Germany and those who arrived in Germany up to five years of age (*p* = 0.543). Furthermore, there was no significant interaction between individuals with Turkish-European lineage and the ethnic minority individuals with other heritage/ancestry (*p* = 0.366).

### Mediation analysis

The total effect of perceived social status mobility on pACC volume was significant (*c* = −0.0050, CI_(95)_ = [−0.00915, −0.0009]) and the bias-corrected confidence intervals of the indirect effect did not contain zero, indicating that the null hypothesis of the absence of an indirect effect had to be rejected (a*b = -− 0.0012, CI_(95)_ = [− 0.0033; − 0.0001]). After the introduction of the mediator, the direct effect between perceived social status mobility scores and pACC GM volume was non-significant (*c’* = −0.0039, CI_(95)_ = [ −0.0079, 0.0002]). This observation supports the hypothesis of a significant indirect effect of social status mobility on pACC volume through chronic stress. The extended mediation model with an additional moderator variable (i.e., German vs. ethnic minority group, acting on the “a path”) did not provide any evidence for a significant influence of ethnic background on the indirect relationship between social mobility, chronic stress, and pACC volume (a*b = −0.0006, CI_(95)_ = [−0.0038; 0.0012]). Percent mediated was 0.2316 (23.16%). This observation suggests that chronic stress mediates the relationship between social status mobility and pACC volume independent of ethnic background.

## Discussion

Social status differences in early life reflect on brain structure and neural functioning in adulthood [46], likely through the accumulation of multiple environmental risk factors that operate at the individual, family and neighborhood level [47] and exert a negative influence on the developing human brain [48]. Many of these risk factors may plausibly converge on increased social stress exposure during upbringing [49], a concept that has been linked to neural alterations in key structures for the regulation of negative emotion and stress, in particular in pACC [30, 32–35]. Meanwhile, although measures of social standing have been related to gray matter volume in pACC [29–31], the potential effects of upward social mobility on brain morphology have not yet been examined.

In this study, we aimed to evaluate the potential effects of perceived upward social mobility on pACC structure and probe the role of chronic stress as a plausible intermediate in this relationship. We studied two relatively large groups of native German and ethnic minority individuals with comparable levels of education, income, perceived social support and social standing in adulthood. Since our ethnic minority sample experienced notably more upward changes in social status from childhood to young adulthood, the investigation of these populations offered the opportunity to examine different degrees of social mobility in the absence of other current demographic and socio-environmental differences that may reflect on brain structure.

Our study provides several interesting outcomes. We detected a significant group-dependent interaction effect of perceived social mobility on pACC gray matter volume, which was driven by a significant negative association in the ethnic minority individuals and the absence of such an association in the German participants. This finding was not explained by several variables implicated in pACC morphology including perceived social standing [30], education [29] and urban upbringing [35] since our groups were either balanced, or we accounted for, these and other factors. Additional sensitivity analysis showed that our results were neither influenced by 1) ethnic minority subgroup nor 2) individuals that came to Germany up to the age of five years.

In addition to social environmental risk, reduced pACC volume has been repeatedly linked to clinical [50–52] and subclinical [53, 54] psychiatric symptom manifestations in the mood-psychosis spectrum. These findings support the notion that healthy descendants of first-generation immigrants with a higher perceived upward social mobility in early life display a brain volumetric signature suggestive of a higher vulnerability for mental illness. In line with this interpretation, epidemiological studies provided consistent evidence for an increased risk for schizophrenia and other psychiatric disorders among immigrants [55] and of functional and structural alterations in pACC in ethnic minorities [32, 34]. The clear persistence of the elevated risk for mental health in their immediate descendants prompted researchers to conclude that post-migration environmental adversity, in particular social experiences related to having an ethnic minority group position in society, are responsible for this phenomenon rather than pre-migration or migration experiences per se [13, 56].

Among these adverse experiences, psychological distress related to perceived racial discrimination is a recognized and a strong predictor of poor mental health in minority ethnic groups [57]. In line with this, we detected a significant mediation of the relationship between perceived social status mobility and pACC volume by perceived chronic stress, a variable that itself was significantly correlated with perceived discrimination in our migrant group. Although our cross-sectional data cannot support causal inferences, the proposed brain morphometric marker for psychiatric vulnerability in pACC may thus be a consequence of stress-related exposures that are inherent to upward social mobility in ethnic minority individuals, including, but not necessarily limited to, perceived discrimination of the own person and ethnic group. Indeed, achieving a higher economic and social status makes upwardly mobile ethnic minorities more likely to stand out relative to their environment of origin, which might subject them to discriminatory experiences [58] above and beyond those shared by all ethnic minority group members. This matches previous findings of upward mobile ethnic minorities reporting higher levels of discrimination experiences than their non-upwardly mobile ethnic minority peers [58], and second-generation migrants reporting higher levels of perceived discrimination compared to first-generation migrants, even given their higher educational level [59].

Beyond ethnic discrimination, moving up the social-economic ladder is an especially strenuous process for ethnic minorities bearing other challenges. Social mobility typically increases the social distance from the original ethnic networks [60], thereby diminishing a crucial source of social support and social embeddedness [16]. This development can foster a sense of social isolation [61] and a destabilizing view of identity that is detrimental to well-being [60]. Furthermore, greater upward social mobility in early life is inherently coupled to a lower childhood social-economic status. This societal disadvantage implicates that individuals have to work with limited social and financial capital in pursuits of goals, which can heighten chronic stress levels and lower life satisfaction and happiness [62]. Notably, low childhood social-economic status is linked to increased chronic stress [63] and brain developmental alterations [31, 46] independent of heritage [48]. However, achieving higher social attainment with more limited resources is likely particularly stressful for the children of immigrants, who experience diminished returns [64] while aiming to accommodate the relatively high educational expectations and aspirations of their parents [65]. We thus believe that, in addition to perceived discrimination, several other challenges that are unaccounted for may have plausibly converged on increased stress exposure during upbringing in our ethnic minority group, thereby contributing to the association between higher upward social mobility, higher perceived chronic stress and lower pACC volume.

The intimate link between social upward mobility and increased chronic stress exposure in ethnic minorities is supported by many studies [62] including studies in the United States reporting higher stress hormone levels [66] higher levels of accelerated epigenetic aging and poorer physical health among upwardly mobile African American adolescents compared to their non-mobile ethnic minority peers [67, 68]. These data are in accordance with the detected volume reduction in pACC in our social upwardly mobile minority individuals. Notably, pACC is a higher order node of the neural emotion and stress regulatory system and is strongly interconnected with other paralimbic brain regions including the amygdala, hippocampus and lower brain regions involved in the generation of adaptive neuroendocrine and autonomic responses to stressful experiences (e.g., hypothalamus, midbrain, and brainstem). Earlier studies in humans [29–31, 34–36] and animals [27, 69, 70] indicate that chronic stress exposure facilitates structural cellular remodeling in pACC, likely via negative feedback effects of glucocorticoids on the neural stress regulatory system [71]. Our data extend these findings by linking upward social mobility in ethnic minorities to a structural neural correlate in pACC, thereby relating the structural integrity of this region to perceived chronic stress and discrimination experiences in ethnic minorities.

Our study has several limitations to consider. First and foremost, the inclusion of native, rather non-mobile, and minority, rather mobile groups in this study did not allow us to disentangle the effects of ethnic minority status and upward social mobility. As a consequence, we cannot exclude the possibility that the relationship between social mobility, chronic stress and pACC volume is only valid for the minority group, for example, due to ethnicity-specific factors such as genetic population stratification effects. Alternatively, the absence of a main effect of social mobility on pACC volume in our majority ethnic group may plausibly relate to the restricted variance in the upward social mobility measure in the German participants. We favor the second interpretation, since *a)* the ethnic background of our minority group was diverse and *b)* the current literature does not suggest a genetic link between early social adversity, chronic stress exposure and related alterations in brain developmental trajectories [37, 48, 63]. This implicates that albeit of the lack of a direct association between social mobility and pACC volume in the majority ethnic group, the German participants have contributed to the detected indirect association via chronic stress. Taken together, these data lead us to believe that the detected link between upward social mobility and the described brain structural vulnerability marker for developmental stress and mental illness may be particularly pronounced, but is likely not exclusive, to ethnic minorities. Notably, this hypothesis requires further inquiry in studies including majority ethnic individuals from more disadvantaged social strata with higher upward social mobility. Furthermore, a second obvious limitation of our work is the cross-sectional nature of the study design, which prevents us from studying causal influences. Although complex social phenomena such as upward social mobility do not lend themselves to experimental manipulation, large longitudinal epidemiological cohort designs or randomized interventional studies with neuroimaging and ecological momentary assessments of perceived stress levels in real life are merited in future to address the underlying causal mechanisms. As a third limitation, our results might alternatively reflect the exposure to neurodevelopmental distress specific to disadvantaged social status very early in life that affects obstetric outcomes and childhood development. In this case, our results would not illustrate the effect of upward social mobility but rather the impact of environmental determinants during critical early neurodevelopmental windows that carry over/persist into adulthood. Even though our data cannot pinpoint one interpretation, our mediation analysis that included chronic stress levels argues for effects that may occur later in life as well. However, one cannot exclude that the experience of higher chronic stress levels originates in lower stress resilience that roots in early exposure to neurodevelopmental distress.

In conclusion, this study provides evidence for an association between higher upward social mobility and reduced pACC volume in ethnic minority individuals, a key neural node implicated in mechanisms sustaining the development and persistence of stress-related mental disorders. It further highlights the crucial role of perceived chronic stress in mediating the relationship between social mobility and pACC volume. These data point to a biological signature of the “allostatic costs” of socioeconomic attainment in socially disadvantaged upwardly mobile individuals, a potential stress-related mechanism that may be independent of ethnic heritage. Our data add further insights to an expanding literature highlighting pACC as a key neural convergence site for multiple environmental risk factors and social stress exposures during upbringing, which include, but are likely not limited to, adverse influences such as racial discrimination that are abundantly experienced by ethnic minorities. Our interpretation is in line with existing theories assuming an interference of early social risk exposures with the ongoing maturation of the neural stress regulatory system, an impairment that may plausibly contribute to the increased psychiatric vulnerability seen in socially disadvantaged populations, including ethnic minorities.

## Supplementary Information

Below is the link to the electronic supplementary material.

Supplementary file1 (DOCX 504 KB)

## Declarations

### Availability of data and material

The code used for the analysis is available within the freeware SPM package (https://www.fil.ion.ucl.ac.uk/spm/). Data sharing is handled by the corresponding author and subject to GDPR restrictions.

### Code availability

Standard SPM protocols were implemented.
