# Supplementary material for: Brain structural correlates of upward social mobility in ethnic minority individuals
Source: Soc Psychiatry Psychiatr Epidemiol. 2021 Aug 12;57(10):2037–47. doi: 10.1007/s00127-021-02163-0 (PMC9477908; doi:10.1007/s00127-021-02163-0)
Supplement: Supplementary file 1 — Supplementary file1 (DOCX 504 KB) [file 127_2021_2163_MOESM1_ESM.docx]

**<<<Supplements**

**
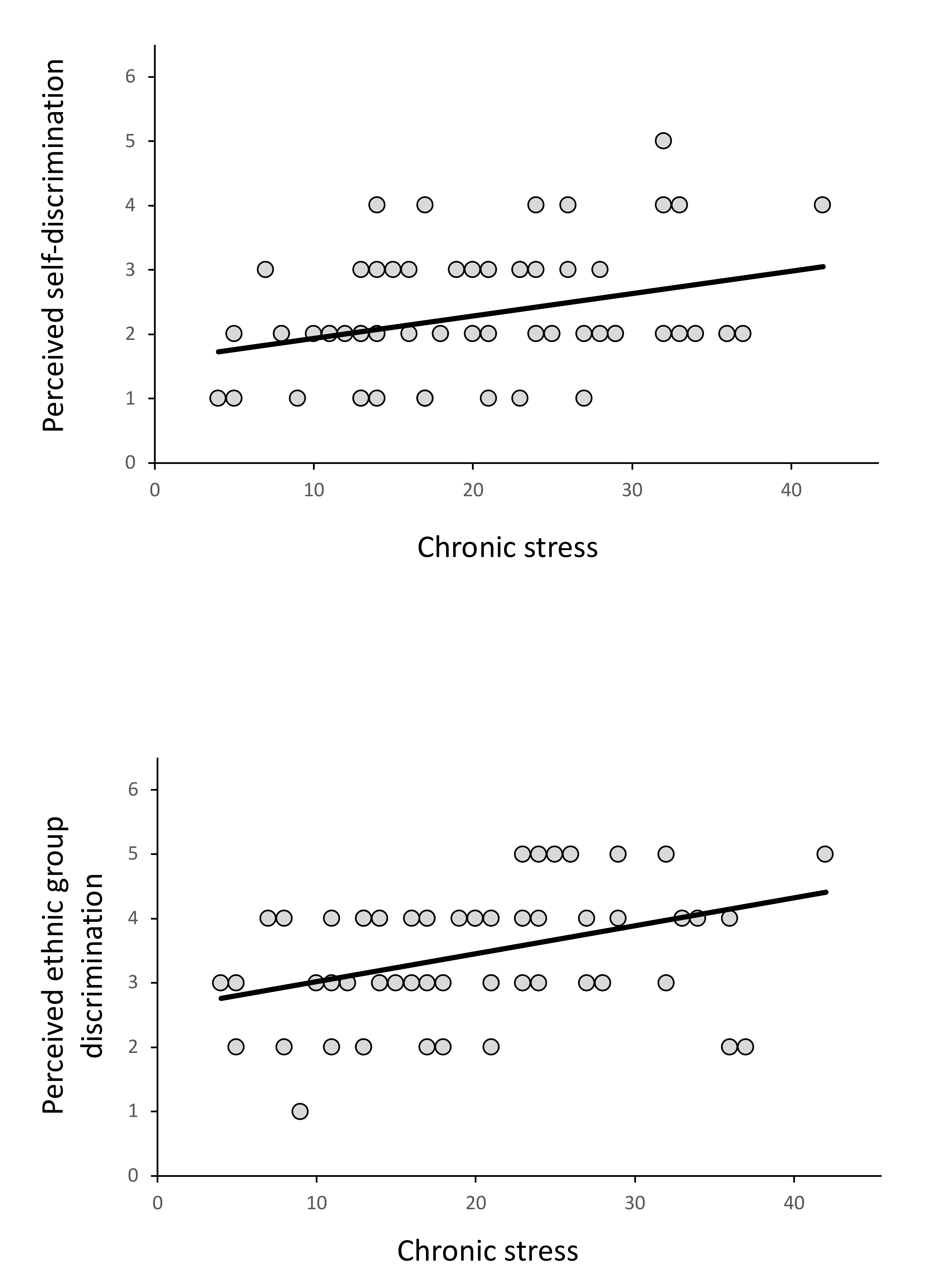
**

**Supplementary Figure a. and b.:** Significant positive correlation between higher chronic stress discrimination experiences in individuals with ethnic minority background. Perceived self (*β* = .29, *p* = .017) and ethnic group discrimination (*β* = .39, *p* = .001).

**Supplementary Table 1: Mediation analysis result**

|  |  | ***M*** (perceived chronic stress) | | |  | ***Y*** (pACC volume) | | |
| --- | --- | --- | --- | --- | --- | --- | --- | --- |
|  |  | Coefficient | SE | *p* |  | Coefficient | SE | *p* |
| ***X*** (perceived social mobility) | *a_1_* | 0.8038 | 0.3816 | .0378 | *c’* | -0.0039 | 0.0021 | .0647 |
| ***M*** (perceived chronic stress) |  | - | - | - | *b_1_* | -0.0014 | 0.0005 | .0084 |
| ***C_1_*** (age) | *a_2_* | -0.5473 | 0.2099 | .0150 | *b_2_* | -0.0035 | 0.0012 | .0045 |
| ***C_2_*** (sex) | *a_3_* | -2.5837 | 1.6872 | .1289 | *b_3_* | -0.0113 | 0.090 | .2153 |
| ***C_3_*** (education) | *a_4_* | 0.1464 | 0.7713 | .8498 | *b_4_* | 0.0063 | 0.0041 | .1261 |
| ***C_4_*** (employment) | *a_5_* | -0.2748 | 1.7631 | .8765 | *b_5_* | 0.0042 | 0.0093 | .6498 |
| ***C_5_*** (income) | *a_6_* | 0.0005 | 0.0006 | .3356 | *b_6_* | 0.0000 | 0.0000 | .9069 |
| ***C_6_*** (early urbanicity) | *a_7_* | 0.1364 | 0.1020 | .1844 | *b_7_* | 0.0007 | 0.0005 | .1999 |
| ***C_7_*** (current urbanicity) | *a_8_* | -1.9642 | 1.6306 | .3765 | *b_8_* | -.0094 | 0.0087 | .1999 |
| ***C_8_*** (household size) | *a_10_* | 0.4247 | 0.3925 | .2820 | *b_9_* | -0,0009 | 0.0021 | .6706 |
| **Constant** | *i_M_* | 28.919 | 12.33 | .0210 | *i_Y_* | .6442 | 0.0670 | <.0001 |
|  |  | *R^2^* = .1616 | | |  | *R^2^* = 0.2093 | | |
|  |  | *F*_(8,31 )_ = 2.0767, *p* = .0389  Percent mediated (Indirect effect/total effect): 23,16% | | |  | *F*_(7,32 )_ = 2.5416, *p* = .0092 | | |
